# Sex Differences in the Broad Autism Phenotype: Insights from the Australian Biobank

**DOI:** 10.1007/s10803-024-06466-4

**Published:** 2024-07-18

**Authors:** Blaise Di Mento, James Rufus John, Antonio Mendoza Diaz, Ping-I Lin, Anne Masi, Rachel Grove, Valsamma Eapen

**Affiliations:** 1 Discipline of Psychiatry and Mental health, Faculty of Medicine, UNSW Sydney, Randwick, NSW Australia; 2 Tasmanian Centre for Mental Health Service Innovation, Hobart, TAS Australia; 3 Autism CRC, Long Pocket, Brisbane, QLD Australia; 4 School of Public Health, Faculty of Health, University of Technology Sydney, Ultimo, NSW Australia; 5 Academic Unit of Infant Child and Adolescent Psychiatry Services (AUCS), South Western Sydney Local Health District, ICAMHS, L1 MHC, Liverpool Hospital, Elizabeth Street, Liverpool, 2170 NSW Australia

**Keywords:** Neurodevelopmental disorders, Autism, Broad autism phenotype, Child psychopathology

## Abstract

Examining sub-threshold autistic traits in non-autistic first-degree relatives of individuals on the autism spectrum, known as the Broad Autism Phenotype (BAP), could provide new insights into the associations and familial aggregation of autistic traits. This study was a retrospective cross-sectional study of parents (*n* = 1008), probands with autism (*n* = 613), and unaffected siblings (*n* = 221) of probands with autism. BAP traits were examined by the BAP Questionnaire and Communication Checklist-Adult in parents, Autism Developmental Observation Scale-Second edition in probands, and Social Responsiveness Scale in siblings. Multivariable linear regression analyses were used to investigate the associations of parental BAP traits on autistic traits in probands and unaffected sibling BAP traits. Fathers showed significantly increased aloofness, pragmatic language difficulties, and social engagement problems compared to mothers. Female siblings showed increased difficulties with social cognition compared to male siblings. Adjusted models of the regression analyses showed that all BAP traits in fathers were significantly associated with BAP trait expression in probands with autism. Additionally, all of mother’s BAP traits were significantly associated with unaffected siblings’ BAP trait expression while only fathers’ aloofness and rigidity traits were inversely associated with siblings’ BAP trait expression. Finally, there were significant inverse interactions noted between parent’s BAP traits and their children’s BAP trait expression. This study demonstrated differences in how males and females express BAP traits and also identified differences in parent-child associations by sex, with fathers having a greater effect on their proband children’s expression of BAP traits than mothers.

## Introduction

The Broad Autism Phenotype (BAP) is comprised of social, communication, and behavioural characteristics. BAP features may provide a strategy to understand the complex and heterogenous presentation and genetics of autism (Moreno-De-Luca & Martin, 2021). These symptoms are not intended to index a clinical diagnosis of autism but rather represent a milder form of autistic traits which reflect the genetic liability to autism (Hurley et al., 2007). This is mediated by a variety of genetic mutations that have been linked to autism, making it difficult to understand clinical and genetic relationships which, in turn, may hamper early identification and intervention of children with autism (Geschwind, 2011; Moreno-De-Luca & Martin, 2021). Hence, research into the BAP in first-degree relatives may provide a new lens to better understand the complexity in autism (Pisula & Ziegart-Sadowska, 2015; Rubenstein & Chawla, 2018), as it may help parcel out which components of autism index impairment from those that do not.

Despite being sub-clinical, these BAP traits can still be measured and classified into domains (e.g., social, communication, and behavioural), although significant diversity exists in the way these traits are expressed. This reflects the putative genetic underpinnings that underlie the manifestation of BAP traits in first degree relatives (Gerdts & Bernier, 2011; Kellerman et al., 2019; Losh et al., 2008; Pisula & Ziegart-Sadowska, 2015). Since the BAP does not represent a formal diagnosis, there is no standardized criteria for measuring BAP traits in individuals. This has led to the creation of multiple survey measures which define BAP traits somewhat differently. For example, the BAP Questionnaire (Hurley et al., 2007) includes aloofness, pragmatic language deficits, and rigidity while the Modified Personality Assessment Scale includes overly conscientious, anxiety, and hypersensitivity (Tyrer, 1988). Given the heritable components of autism, studies of sub-clinical markers such as the BAP may be beneficial in differentiating those components of complex disorders that are not associated with clinical severity (Burmeister et al., 2008). Thus, understanding the presentation and familial aggregation of BAP traits may further an understanding of the underlying biological pathways in autism (Losh et al., 2008), as it may help the field in differentiating the heritability of common aspects of autism from that of rare variants associated with specific forms of impairment, as seen in the recent study by Antaki et al. (2022).

Despite ongoing research into the BAP, there has been little work in characterizing the differences in BAP presentation by sex. Most studies have focused on a broad conceptualization of phenotypic differences, demonstrating a greater severity of BAP traits in fathers most notably in social and communication domains as measured by a range of instruments (Markiewicz et al., 2020; Maxwell et al., 2013; Schwichtenberg et al., 2010; Sucksmith et al., 2013). Such studies looked at vaguely defined traits including aloofness, empathy, and social communication. However, evaluating studies which investigated the differences between specific traits have yielded contrasting results. While most studies are in agreeance about increased aloofness in fathers (Maxwell et al., 2013; Seidman et al., 2011; Sucksmith et al., 2013), there are mixed results regarding pragmatic language deficits and rigidity with some studies suggesting increased difficulties in fathers (Davidson et al., 2014; Whitehouse et al., 2010) while others found no sex differences (Klusek et al., 2014; Losh et al., 2008; Seidman et al., 2011). The general lack of consensus on sex differences in parental traits is likely the result of the use of differing measures to capture BAP expression in parents as well as diversity in methodology and sample size. Additionally, previous studies have largely neglected to control for the normal distribution of these traits in the general population as males generally express a greater severity of these traits (Sasson et al., 2013a; Hurley et al., 2007; Whitehouse et al., 2010).

Sex differences specifically related to the BAP of siblings of autistic individuals are generally poorly understood, as they have tended not to be the focus of research. Despite this, much is known regarding sex differences in the domains of language and communication that are purportedly described by the BAP, with young girls tending to out-perform males in the development of language and communication skills (Sørlie et al., 2021). This has recently been the source important debates regarding the under-representation of women with autism and their masking of autistic traits by virtue of better language skills (Wood-Downie et al., 2021b; Wood-Downie et al., 2021a). Male and female siblings have shown differing patterns in social attention as reflected by their visual attention to people displaying emotion (Kleberg et al., 2019). In this, male siblings focussed more on the mouth of facial expressions in comparison to female siblings. Another study used the ‘Autism Quotient’ to measure differences in siblings which is an instrument measuring autistic traits of communication, social skills, imagination, attention to detail, and attention switching (Baron-Cohen et al., 2001). In this study, siblings of individuals with autism were compared to individuals that did not have a sibling with autism (controls). While there were no differences in adult male and female siblings, female adolescent siblings showed greater autistic traits than female controls in all domains of the autism quotient except for ‘Imagination’. In contrast, male adolescent siblings showed greater autistic traits in the ‘Communication’ subscale compared to male controls. Thus, there seems to be a phenotypical difference in expression of autistic traits by sex.

The most definite attempt at characterizing these differences in siblings occurred in a study by Jussila et al. (2015) which found increased difficulties in social cognition and social motivation in male siblings as measured by raw scores on the parent-completed Social Responsiveness Scale (SRS). However, given the differing expression of autistic traits by males and females in the population, as discussed above, there is a need to explore these differences in a manner that controls for innate sex-specific expression of the BAP. Considering the expression of the BAP in parents and siblings together may establish sex-specific patterns which may help in future assessment and exploration of the BAP in first-degree relatives.

Furthermore, there is limited research on differences of the BAP in terms of familial aggregation, that is, how traits cluster in a family. Several studies have focused broadly on the relationship between parental BAP symptoms and BAP symptoms in children (De La Marche et al., 2015; Maxwell et al., 2013; Rubenstein et al., 2019) but only some have looked at the relationship between specific BAP traits in parents with those in their children (Hasegawa et al., 2015; Klusek et al., 2014; Nayar et al., 2021). These studies generally agree that BAP traits in mothers are associated with social difficulties in children while those in fathers are more associated with behavioural difficulties in children. However, these studies did not consider the effect of paternal and maternal BAP traits on male and female children separately. Additionally, these studies have only investigated the relationship between BAP traits in parents and autistic traits in children with autism. It is also important to investigate how BAP traits in parents relate to those in unaffected siblings, particularly whether traits expressed in mothers and fathers are passed on equally to male and female children. The authors are mindful of historical claims conferring blame to parents for the autistic traits in their children, which we reject. Rather, the approach pursued in this article aims to show how non-clinical features are passed-down between generations.

While there are clear sex differences in the presentation of autism, causing under-diagnosis and misdiagnosis of females, it is unclear whether the traits that make up the BAP also vary in their presentation (Loomes et al., 2017). Additionally, it is unclear how specific BAP traits cluster in family units (Nayar et al., 2021; Sasson et al., 2013b). Hence, this study aims to address the research question of whether parental BAP traits are associated with children’s BAP traits and if there are any variations by the diagnosis of autism and by sex.

## Methods

### Participants

Data was sourced from the Australian Autism Biobank (AAB), Australia’s largest collection of data and samples from autistic children and their families. Detailed phenotypic data was taken from three groups: (1) children with autism (probands), children aged 2–17 years who received a clinically confirmed diagnosis of autism, (2) parents of children with autism, and (3) siblings of children with autism who did not themselves meet the criteria for autism. For the purpose of this study, we included 613 probands (483 males), 1008 parents who completed the BAP Questionnaire (BAPQ) at baseline (435 fathers), and 221 siblings (107 males). A more comprehensive description of recruitment and clinical phenotyping for the AAB is seen in Alvares et al. (2018).

### Measures

Proband child assessments included the Autism Diagnostic Observation Schedule-second edition (ADOS-2) and the Wechsler Intelligence Scale for Children, Fifth Edition (WISC-V) whereas parental assessments comprised the Broad Autism Phenotype Questionnaire (BAPQ) and Communication Checklist-Adult (CC-A). Sibling assessment measures included the WISC-V and SRS assessments. Further details about the data collection are reported elsewhere (Alvares et al., 2018).

### Autism Diagnostic Observation Schedule-Second Edition (ADOS-2)

The ADOS-2 is a semi-structured interview used to measure language and communication skills, social interaction, and stereotypical and restricted behaviours in autistic individuals (Lord et al., 2000). The use of four different modules, based on the subject’s language skills allows for analysis across a wide range of presentations. A calibrated severity score (CSS) was used to compare overall autism severity between modules (Gotham et al., 2009). In this study, there is acceptable level of internal consistency of the scale (Cronbach’s alpha = 0.71).

### Wechsler Intelligence Scale for Children, Fifth Edition (WISC-V) Assessment

The (WISC-V) (Wechsler, 2014) is a widely employed assessment tool designed to measure the cognitive abilities of children aged 6 to 16 years. This comprehensive test consists of 16 subtests organized into five main indices, including the Verbal Comprehension Index, Fluid Reasoning Index, Visual Spatial Index, Working Memory Index, and Processing Speed Index. These indices collectively contribute to the Full Scale IQ (FSIQ) score, providing a nuanced understanding of a child’s intellectual strengths and weaknesses. The Cronbach’s alpha of the WISC-V in this study was 0.94, demonstrating excellent internal consistency.

### Social Responsiveness Scale-2nd Edition (SRS)

The SRS is a 65-item parent-completed questionnaire that measures a child’s ability to reciprocate emotion and conversation in a social context (Constatino & Gruber, 2012). In the school-age and preschool-age forms, items are mapped onto five subscales of social awareness, social cognition, social communication, social motivation and restricted interests and repetitive behaviours (RRBs). There are also two total scores of social and communication impairments (SCI) and restricted interests and repetitive behaviors (RIRB). T-scores were used in this analysis which are calculated separately for males and females to account for normal sex differences. These scores were used to measure the expression of BAP symptoms in siblings without a diagnosis of autism. The Cronbach’s alpha of the SRS in this study was 0.90, demonstrating excellent internal consistency.

### Broad Autism Phenotype Questionnaire (BAPQ): Self Report

The BAPQ is a 36-item questionnaire which measures personality and language characteristics considered key features of the BAP from a research perspective, i.e., aloofness, rigidity, and pragmatic language deficits (Hurley et al., 2007). Individuals are asked how often a statement applies to them (e.g. I act very set in my ways) with answers on a 6-point scale ranging from very rarely (1) to very often (6). The BAPQ includes reverse-scored items which were reversed to create subscale totals for the three key domains of the BAPQ, i.e., aloofness, pragmatic language deficits, and rigidity. The Cronbach’s alpha of the BAPQ in this study was 0.91, demonstrating excellent internal consistency.

### Communication Checklist-Adult (CC-A)

The CC-A is an informant-reported questionnaire about social and communicative behaviours in everyday situations. It measures BAP traits by three scales pertaining to language structure, pragmatic skills, and social engagement (Whitehouse et al., 2010). It was shown to be a valid measure of social and communicative difficulties and has demonstrated some sensitivity in measuring BAP traits (Whitehouse et al., 2010). Z-scores were used in analysis to account for normal sex differences in these traits where a higher z-score corresponds to increased social or behavioural difficulties. The Cronbach’s alpha of the CC-A in this study was 0.96, demonstrating excellent internal consistency.

### Data Analysis

Data analysis was conducted in IBM SPSS Statistics Version 26.0 (IBMCorp, 2019) with two-tailed significance set at *p* value of 0.05. Variations in sociodemographic data as well as clinical assessments of parental and children were evaluated by independent samples t-tests and Pearson’s chi-square analyses. In order to identify and address potential outliers in the dataset, we employed a comprehensive approach by visual inspection of the data through box plots and scatter plots, which allowed us to visually identify any data points that deviated significantly from the overall pattern. Subsequently, we employed statistical methods such as interquartile range to quantitatively identify outliers based on their deviation from the mean or median.

A univariable linear regression analysis was conducted to determine the independent association between parental and children’s BAP traits. Additionally, a multivariable linear regression analysis examined the relationship between parental BAPQ scores with proband ADOS severity scores as well as siblings SRS total scores. The regression analyses were adjusted for covariates such as child’s age and sex. Pearson’s correlations tests were performed to test for multicollinearity before entering them into the regression models. Further, interaction analysis was conducted to understand the differing effect of parental BAP traits on male and female probands and siblings separately.

## Results

### Demographics

The demographic characteristics of parents and children are presented in Tables 1 and 2. Fathers (*M* = 41.5, *SD* = 7.2) were significantly older than mothers (*M* = 38.8, *SD* = 6.1), *t*(812) = 6.16, *p* < 0.001. Findings of the chi-square tests showed significant difference in the level of education between fathers and mothers where a higher proportion of mothers had university degree, *χ2* (2, *N* = 965) = 10.12, *p* = 0.006 (Table 1). For children, both male probands (*M* = 6.9, *SD* = 3.9) as well as siblings (*M* = 7.6, *SD* = 4.3) were younger than female probands (*M* = 7.3, *SD* = 3.9) and siblings (*M* = 8.7, *SD* = 4.1) but these findings were not statistically significant (Table 2).

**Table 1:** Demographic characteristics of parents

Characteristics	Total (*N* = 1008) M (SD) or *n* (%)	Fathers (*N* = 435) M (SD) or *n* (%)	Mothers (*N* = 573) M (SD) or *n* (%)	*p*-value
Age in years	39.9 (6.7)	41.5 (7.2)	38.8 (6.1)	**< 0.001**
Highest level of education				**0.006**
*12 years of school or less*	218 (21.6)	94 (21.6)	124 (21.6)	
*Trade*,* Technical Certificate V*	266 (26.4)	136 (31.3)	130 (22.7)	
*University Degree*	481 (47.7)	188 (43.2)	293 (51.1)	
*Unknown*	43 (4.3)	17 (3.9)	26 (4.5)	
Ethnic Background				0.221
*Caucasian*	792 (78.6)	353 (81.1)	439 (76.6)	
*Aboriginal and Torres Strait Islander*	9 (0.9)	5 (1.1)	4 (0.7)	
*Asian*	109 (10.8)	43 (9.9)	66 (11.5)	
*Maori or Pacific Islander*	11 (1.1)	4 (0.9)	7 (1.2)	
*Other*	64 (6.3)	20 (4.6)	44 (7.7)	
*Unknown*	23 (2.3)	10 (2.3)	13 (2.3)	
Children from previous relationship				0.173
*No*	901 (89.4)	378 (89.6)	523 (91.3)	
*Yes*	89 (8.8)	44 (10.4)	45 (7.8)	
*Missing/Unknown*	18 (1.8)	13 (3.0)	5 (0.9)	

** *p* < 0.001, M – mean, SD – Standard Deviation

**Table 2:** Demographic characteristics of probands and siblings

Characteristics	Total M (SD) or *n* (%)	Probands			Siblings		
		Male (*N* = 483) M (SD) or *n* (%)	Female (*N* = 130) M (SD) or *n* (%)	*p*-value	Male (*N* = 107) M (SD) or *n* (%)	Female (*N* = 114) M (SD) or *n* (%)	*p*-value
Age	7.3 (3.9)	6.9 (3.9)	7.3 (3.9)	0.362	7.6 (4.3)	8.7 (4.1)	0.131
Number of other children with autism in Family				0.999			0.895
*None*	562 (67.4)	330 (68.3)	89 (68.5)		69 (64.5)	74 (64.9)	
*One*	207 (24.7)	107 (22.2)	29 (22.3)		34 (31.8)	37 (32.5)	
*More than one*	65 (7.9)	46 (9.5)	12 (9.2)		4 (3.7)	3 (2.6)	
Language spoken at home				0.456			0.183
*English*	761 (91.2)	454 (94.0)	121 (93.1)		88 (82.2)	98 (86.0)	
*Other*	31 (3.7)	22 (4.6)	6 (4.6)		0 (0.0)	3 (2.6)	
*Missing/Unknown*	42 (5.1)	7 (1.4)	3 (2.3)		19 (17.8)	13 (11.4)	
Annual family income				0.664			0.284
*Up to $40*,*000*	46 (5.5)	32 (6.6)	10 (7.7)		2 (1.9)	2 (1.8)	
*$40*,*000 to $104*,*000*	253 (30.3)	168 (34.8)	44 (33.8)		23 (21.5)	18 (15.8)	
*More than $104*,*000*	269 (32.3)	182 (37.7)	41 (31.5)		18 (16.8)	28 (24.6)	
*Missing/Unknown*	266 (31.9)	101 (20.9)	35 (26.9)		64 (59.8)	66 (57.9)	

M -mean; SD - Standard Deviation

### Parental Sex Differences in BAP Traits

The differences in the BAP traits in fathers and mothers are presented in Table 3. Fathers demonstrated significantly higher BAPQ scores in the traits of aloofness (*Mean difference (MD)* = 0.32, *Standard Error (SE)* = 0.06, *t*(1006) = 5.64, *p* < 0.001), pragmatic language (*MD* = 0.17, *SE* = 0.05, *t*(1006) = 3.60, *p* < 0.001), and rigidity (*MD* = 3.08, *SE* = 0.87, *t*(1006) = 2.19, *p* = 0.028) than mothers. Additionally, mothers demonstrated significantly higher scores of difficulties in language structure (*MD* = 0.10, *SE* = 0.04, *t*(871) = 2.04, *cohen’s d* = 0.14, *p* = 0,041), pragmatic language (*MD* = 0.27, *SE* = 0.06, *t*(906) = 4.52, *cohen’s d* = 0.30, *p* < 0.001), and social engagement (*MD* = 0.31, *SE* = 0.09, *t*(900) = 3.52, *cohen’s d* = 0.23, *p* < 0.001) compared to fathers.

**Table 3:** Differences in BAP traits in fathers and mothers

BAP Traits	Fathers M (SD)	Mothers M (SD)	Mean Difference (SE)	t Statistic	*p*-value
BAPQ Raw Score					
*Aloofness*	3.02 (0.90)	2.69 (0.89)	0.32 (0.06)	5.64	< 0.001
*Pragmatic Language*	2.54 (0.74)	2.37 (0.72)	0.17 (0.05)	3.60	< 0.001
*Rigidity*	3.08 (0.87)	2.96(0.86)	0.12 (0.06)	2.19	0.028
CC-A Z-Score					
*Language Structure*	-0.59(0.76)	-0.69(0.77)	0.10 (0.04)	2.04	0.041
*Pragmatic Difficulties*	-0.33 (0.84)	-0.60(0.98)	0.27 (0.06)	4.52	< 0.001
*Social Engagement*	-0.70(1.24)	-1.01(1.40)	0.31 (0.09)	3.52	< 0.001

BAPQ - Broad Autism Phenotype Questionnaire; CC-A -Communication Checklist-Adult; M – mean; SD - standard deviation; SE – Standard Error

### Sex Differences in Proband and Siblings’ BAP Traits

The differences in the BAP traits in probands and siblings by sex are presented in Table 4. Female siblings recorded higher t-scores in the social cognition domain of the SRS compared to male siblings (*MD* = -6.07, *SE* = 3.08, *t*(30) = -1.97, *cohen’s d* = 0.68, *p* = 0.050) as seen in Table 4. There were no other differences by sex.

**Table 4:** Differences in BAP traits in proband and siblings

BAP Traits	Proband				Siblings			
	Male M (SD)	Female M (SD)	Mean Difference (SE)	*p*-value	Male M (SD)	Female M (SD)	Mean Difference (SE)	*p*-value
WISC-V FSIQ Score	86.21 (22.31)	85.67 (23.97)	0.54 (3.38)	0.878	100.33 (13.82)	103.25 (12.48)	-2.92 (2.35)	0.218
ADOS-2 CSS score	6.40 (2.44)	6.58 (2.23)	-0.19 (0.24)	0.435	-	-	-	-
Social Awareness	-	-	-	-	49.25 (9.93)	51.82 (14.89)	-2.57 (4.46)	0.569
Social Cognition	-	-	-	-	43.48 (6.93)	49.55 (10.46)	-6.07 (3.08)	**0.050**
Social Communication	-	-	-	-	45.29 (8.03)	50.64 (14.79)	-5.35 (4.79)	0.284
Social Motivation	-	-	-	-	48.52 (6.68)	51.40 (7.25)	-2.88 (2.64)	0.284
RIRB	-	-	-	-	48.67 (9.58)	53.64 (16.02)	-4.97 (4.51)	0.279
SCI	-	-	-	-	45.43 (7.22)	51.55 (13.40)	-6.12 (3.62)	0.102

M – Mean; SD – Standard Deviation; SE – Standard Error; ADOS-2 - Autism Diagnostic Observation Schedule-Second Edition; CSS – calibrated severity score; WISC-V - Wechsler Intelligence Scale for Children Fifth Edition; FSIQ - Full Scale IQ; RIRB - restricted interests and repetitive behaviors; SCI - social and communication impairments

### Familial Aggregation

Child age was found to be significantly correlated with ADOS-2 subscale scores of SA (*r* = 0.066, *p* = 0.03) and RRB (*r* = 0.157, *p* < 0.001) domains. Multiplex status also predicted child scores in SA (*p* = 0.006) and RRB (*p* < 0.001) domains. There was no association between maternal BAPQ scores (*p* > 0.150) or paternal BAPQ scores (*p* > 0.079) with proband ADOS-2 calibrated severity scores.

Findings of the multivariable regression models are presented in Tables 5 and 6. In terms of the relationship between parental BAP traits and probands overall autism severity, the adjusted models showed that all of father’s BAP traits such as aloofness (*b* = 0.29, *SE* = 0.15, *p* = 0.050), pragmatic language (*b* = 0.57, *SE* = 0.19, *p* = 0.002), and rigidity (*b* = 0.31, *SE* = 0.15, *p* = 0.045) were significantly associated with autism severity in the proband children with autism. Additionally, there were significant inverse interaction between father’s BAP traits and female probands (Table 5).

**Table 5:** Influence of parental BAP traits on proband’s ADOS-2 CSS scores

Predictors	Unadjusted model		Adjusted model	
	B (SE)	*p*-value	B (SE)	*p*-value
Aloofness – Father	0.11 (0.09)	0.210	0.29 (0.15)	**0.050**
Pragmatic Language – Father	0.16 (0.10)	0.126	0.57 (0.19)	**0.002**
Rigidity – Father	0.13 (0.09)	0.133	0.31 (0.15)	**0.045**
Aloofness - Mother	0.10 (0.13)	0.446	0.05 (0.24)	0.851
Pragmatic Language – Mother	0.23 (0.16)	0.151	0.22 (0.26)	0.403
Rigidity – Mother	0.27 (0.14)	**0.050**	0.27 (0.25)	0.284
Female sex * Aloofness – Father	-0.39 (0.18)	**0.034**	-0.38 (0.18)	**0.039**
Female sex * Pragmatic Language – Father	-0.64 (0.22)	**0.004**	-0.60 (0.22)	**0.006**
Female sex * Rigidity – Father	-0.35 (0.18)	0.060	-0.36 (0.18)	**0.050**
Female sex * Aloofness - Mother	0.12 (0.28)	0.680	0.15 (0.29)	0.594
Female sex * Pragmatic Language – Mother	-0.02 (0.33)	0.963	-0.06 (0.33)	0.865
Female sex * Rigidity – Mother	-0.05 (0.30)	0.860	0.01 (0.29)	0.986

Adjusted model – adjusted for child’s age and sex

**Table 6:** Influence of parental BAP traits on unaffected sibling SRS scores

Predictors	Unadjusted model		Adjusted model	
	B (SE)	*p*-value	B (SE)	*p*-value
Aloofness – Father	-2.19 (1.68)	0.199	-5.68 (2.82)	**0.050**
Pragmatic Language – Father	-2.03 (2.07)	0.333	-7.22 (3.96)	0.079
Rigidity – Father	-1.81 (1.75)	0.308	-5.99 (2.94)	**0.050**
Aloofness - Mother	3.99 (2.30)	0.091	9.96 (3.56)	**0.009**
Pragmatic Language – Mother	3.97 (2.68)	0.147	8.31 (3.91)	**0.043**
Rigidity – Mother	4.79 (2.44)	**0.050**	10.62 (4.25)	**0.019**
Female sex * Aloofness – Father	6.32 (3.57)	0.085	5.83 (3.42)	0.099
Female sex * Pragmatic Language – Father	8.72 (4.84)	0.080	8.43 (4.63)	0.080
Female sex * Rigidity – Father	5.36 (3.69)	0.155	4.99 (3.57)	0.173
Female sex * Aloofness - Mother	-11.32 (5.37)	**0.042**	-13.17 (5.76)	**0.030**
Female sex * Pragmatic Language – Mother	-9.01 (7.08)	0.211	-4.39 (7.98)	0.587
Female sex * Rigidity – Mother	-8.66 (5.60)	0.131	-12.28 (6.20)	**0.050**

Adjusted model – adjusted for child’s age and sex

With regards to the relationship between parental BAP traits and siblings SRS scores, mother’s BAP traits - aloofness (*b* = 9.96, *SE* = 3.56, *p* = 0.009), pragmatic language (*b* = 8.31, *SE* = 3.91, *p* = 0.043), and rigidity (*b* = 10.62, *SE* = 4.25, *p* = 0.019) were significantly associated with siblings’ SRS scores. On the contrary, father’s aloofness (*b* = -5.68, *SE* = 2.82, *p* = 0.050) and rigidity (*b* = -7.22, *SE* = 3.96, *p* = 0.050) were inversely associated with siblings’ SRS scores. Further, the interaction analysis showed significant inverse interaction between mother’s aloofness and rigidity in female probands (Table 6).

## Discussion

This study aimed to investigate sex differences in the BAP by clinical presentation and familial aggregation. Mothers showed increased pragmatic language difficulties and social engagement issues more than fathers. Female siblings also exhibited increased difficulties with social cognition compared to male siblings. While no parental BAP traits were associated with autistic traits in probands, there were associations between parental BAP traits and siblings. All paternal BAP traits were associated with BAP trait expression in siblings, however only maternal pragmatic language difficulties were associated with BAP expression in siblings regardless of sex. Maternal rigidity was associated with BAP traits in female siblings only.

In examining the sex differences in parents, it is seen that the clinical picture of the BAP in mothers is characterized by increased social difficulties. This is consistent with Seidman et al. (Seidman et al., 2011) who reported higher rigidity scores in mothers compared to fathers. It is noteworthy that these authors also found higher aloofness score in fathers compared to mothers. It is possible that aloofness is easier to recognise as an autism related trait and this is more commonly represented in the male phenotype, allowing better identification. Female phenotype on the other hand may be more often characterized by rigidity in the social context and since this is not routinely enquired about or included in assessments, this trait is probably being missed. Nevertheless, it is important to examine this in detail in future studies (Sasson et al., 2013a). Overall, the results of this study showed that while fathers have increased raw BAP scores, mothers have increased BAP scores in social traits when accounting for sex-specific norms. This would suggest that the phenotypical presentation of the BAP in females may be hidden by the normal distribution of these traits in the population which somewhat mirrors the presentation of autism, whereby females have been found to ‘mask’ their symptoms by mimicking their peers (Rynkiewicz et al., 2016).

In relation to sex differences in unaffected siblings, female siblings tended to have a BAP presentation defined by difficulties in social cognition. This, also, mirrors the sex differences observed in autism. Female children with autism have been found to have reduced expression of RBBs (Beggiato et al., 2017; Hiller et al., 2014; May et al., 2016) along with more subtle social and communication impairments (Craig et al., 2019; Evans et al., 2019). This could mean that these undetected behaviours might be driving the sex differences in this group. This idea is strengthened by the findings of Jussila et al. (Jussila et al., 2015), who found increased raw SRS scores in male siblings compared to female siblings in the domains of social cognition and social motivation. Taken with the findings of this study, which used sex-specific t-scores, it is likely that social cognition is a key domain where girls are most adept at ‘camouflaging’ their symptoms. This would explain why male siblings have increased raw scores, but female siblings have increased t-scores. This presents further evidence of the female presentation of the BAP being characterized by increased social difficulties and demonstrates the importance of sex-specific scoring in further studies and assessment of BAP traits in first-degree relatives.

The lack of associations between parents and probands would appear to negate the possibility that parental BAP is important in understanding autism. However, the lack of associations in this study is likely the result of the measures used, rather than a true finding of non-association. The instruments used in parents were designed to detect mild autistic traits across many domains. This is in contrast to the use of the ADOS in probands, which measures diagnostic traits of autism using a 10-point calibrated severity score, which does not appropriately reflect the multi-faceted expression of autism in an individual. Further research in this field, using a more sensitive and multi-faceted tool to measure symptoms in probands, is necessary.

On examining the associations between parent scores and sibling scores, all three paternal BAPQ subscale scores are noted to relate to sibling BAP traits. This would suggest that BAP traits in fathers could be key predictors for determining severity of autistic traits in siblings, which may also extend to probands. This is consistent with previous studies (Jussila et al., 2015) that found increased associations between paternal BAPQ total score and child SRS subscale scores compared to maternal scores. This suggests the possibility of fathers playing a key role in determining child autism phenotype which is an idea explored in the literature (Maxwell et al., 2013; Schwichtenberg et al., 2010). Further studies could examine this relationship in more detail, including how paternal BAP traits are associated with specific BAP traits measured on the SRS, namely social awareness, social cognition, social communication, and social motivation. It is also possible that most autism measures are ascertaining aloofness and related traits which are more commonly seen in male phenotype, while social issues relating to rigidity may be more common in female phenotype, but this is often not routinely ascertained in clinical practice. Given that there was a significant influence of mother’s rigidity on female siblings’ rigidity scores, this may be a specific sex-dependent trait but since this is not included in routinely used autism scales, female members of the family who have these traits are perhaps being missed. The use of more sensitive and multi-faceted tools to measure symptom expression in probands and family members may uncover associations with paternal BAP traits.

Notably, maternal pragmatic language was significant in determining sibling outcome scores. This mirrors research into the effect of mothers of individuals with autism where mother’s pragmatic language difficulties were observed to be important predictors of functioning in children on the autism spectrum (Hasegawa et al., 2015; Nayar et al., 2021). At the time it was suggested that there was a stronger inherited maternal effect for language-related phenotypes in autism, but this has not been replicated in this study given the strong influence of fathers also in this domain. This may have been the result of representing sibling BAP traits as a function of their total score, rather than looking at individual domains on the SRS.

Further, maternal rigidity had differing effects on a siblings’ presentation based on their sex. When only accounting for female siblings, there was a significant influence of mother’s rigidity on their scores. This was a novel finding but contradicted some previous research that has suggested that mothers’ BAP traits are more related to language and communication difficulties in their children (Klusek et al., 2014; Nayar et al., 2021). This specific relationship may be the result of a combination of familial aggregation and expression of the BAP trait that is defined by their sex. Previous research has suggested that RRB traits, including ‘rigidity,’ are difficult to detect in females (McFayden et al., 2019). Therefore, the association between mothers and sisters could be the result of expression of sub-threshold RRB traits that cannot be detected clinically, but are detected by more sensitive tools such as those used in this study. Further research into this area is required, along with replication of these findings in other samples.

Taken together, the BAP appears to vary by sex in terms of prominent traits and familial aggregation. While challenges remain in BAP identification and assessment, sex-specific norms can be used to highlight increased social difficulties in mothers and female siblings of individuals with autism. Being able to correctly identify these BAP features in first-degree relatives may be useful in the context of developing strategies to cluster autism by genetic risk as determined by BAP presentation in relatives. The strong association between paternal and sibling score suggests that fathers do have a key role in familial aggregation of sibling traits. However, in examining females, a group often misdiagnosed given the differing phenotypic traits, it may be that select BAP traits in their mothers, i.e., rigidity, may be useful in identification and management. Importantly, these associations do not point to a mode of heritability for their children’s autistic traits but rather could be used to better understand the sex dependent heterogeneity in autism presentations.

The BAP could also prove useful as a supplement to traditional methods of ASD diagnosis. This study has demonstrated further evidence for the sex-ratio mismatch in ASD by the increased social impairments found in unaffected female siblings (sisters) but not the male siblings and the strong association observed between mothers’ rigidity scores and the sisters’ social impairment scores. In this light, identifying BAP traits in females could prove useful. Girls on the autism spectrum have been shown to have greater internalizing problems and suffer from social exhaustion as a result of ‘masking’ of symptoms (Hiller et al., 2014; Hull et al., 2020). As such, females who have sub-threshold autistic traits may also be subject to these issues. Identifying BAP traits in this population may be useful from the perspective of providing support and intervention where it is necessary. However, since this was not the focus of the present study, subsequent research would be required to determine the need and implementation of such an approach. Although unrelated to the study aims, another interesting finding is the percentage differences in sex for unaffected siblings based on number of autistic children in the family, which may potentially reflect the female protective effect often observed in autism (i.e., unaffected female siblings seem less likely to have more than one affected sibling in the family, and more likely to be in a family with only one affected sibling).

Strengths of this study included the use of multiple tools to measure the BAP. This allowed a multi-faceted approach to the BAP and added internal validity to sex-differences reported in pragmatic language and social domains. Additionally, the large sample size afforded by accessing the AAB gave this study power to detect differences between males and females.

In terms of limitations, a possible limitation is reporter bias as BAP traits in parents of children with autism may cause them to over-report symptoms in their children. In handling this possibility, this study followed literature suggesting that reporter bias alone is unlikely to underpin the associations found between autistic traits of parents and children. However, recent literature has found that specifically mothers with BAP symptoms may be prone to over-reporting autistic traits in their children (Dovgan et al., 2022). Since it is unknown who completed the SRS, it is difficult to quantify the influence this may have had on the findings. However, given the degree of paternal, rather than maternal, associations with sibling SRS scores it is unlikely this had a major effect although future research with multiple informants to measure symptoms should be employed. Further, the nature of the data is limited by some missingness in certain variables in addition to certain other key risk factors that were not collected, and hence were not accounted for, in our analysis.

Additionally, the differing measures used in the AAB made it difficult to compare findings across groups. Since parents and sibling were not subject to the same BAP measure, the subtle differences could not be distinguished in these groups. Future directions from this study should focus on the role of mothers in the expression of autism traits in children. While this study identified a strong relationship between BAP features such as rigidity in mothers and mild autistic traits in female siblings, there is a need to understand whether there are also relationships between autistic presentations in mothers and daughters with autism. This could be achieved by measuring autistic traits in probands and siblings with a variety of sensitive tools. These findings would add to the current study by either validating or negating the use of parental BAP traits as a way to better understand mild autistic traits and any sex dependent differences in presentations.

## Conclusion

This study assessed the sex differences of the BAP in parents and siblings by looking at phenotypical presentation and familial aggregation. While BAP traits may be magnified in fathers, after accounting for sex differences there is increased rates of aloofness in mothers along with increased difficulties in pragmatic language and social engagement. Female siblings demonstrated greater difficulties in social cognition compared to male siblings. Examining the influence of parental BAP scores on child scores suggested that fathers BAP traits are associated with sibling BAP traits, but mothers and female siblings have a specific association. To date, there has not been characterization of specific phenotypes of the BAP by sex by looking at both parents and siblings of children with autism, nor investigation of the influence of certain BAP subscale scores on child subscale scores by sex. While this study has addressed these points, further research into the role of parental BAP on autism presentation and severity are required.

## Data Availability

Data is available upon formal request to Autism CRC (autismcrc.com.au/biobank).
